# Molecular Identification of *Candida albicans* in Endodontic Retreatment Cases by SYBR Green I Real-time Polymerase Chain Reaction and its Association with Endodontic Symptoms

**DOI:** 10.30476/dentjods.2023.91706.1786

**Published:** 2023-12-01

**Authors:** Ahmad Nouroloyouni, Negar Moghaddam, Sarah Nuroloyuni, Amin Salem Milani, Hamid Reza Yavari, Ali Reza Majidi

**Affiliations:** 1 Dept. of Endodontics, Faculty of Dentistry, Ardabil University of Medical Sciences, Ardabil, Iran; 2 Dept. of Endodontics, Faculty of Dentistry, Tabriz University of Medical Sciences, Tabriz, Iran; 3 Dept. of Pediatric Dentistry, Faculty of Dentistry, Ardabil University of Medical Sciences, Ardabil, Iran; 4 Dept. of Endodontics, Faculty of Dentistry, Qom University of Medical Sciences, Qom, Iran

**Keywords:** *Candida albicans*, Endodontics, hsp60 protein, Polymerase chain reaction

## Abstract

**Statement of the Problem:**

Recent microbiological studies have expressed ever-increasing concerns about *Candida albicans* as a causal factor in the failure of endodontic treatments. Real-time quantitative polymerase chain reaction (qPCR), including the SYBR Green I system, is a technique in which a fluorescent dye is incorporated into the double-stranded DNA that is produced during DNA polymerase activity.

**Purpose:**

This study aimed to determine the relative prevalence of *Candida albicans* in root canals of retreatment cases and its association with endodontic symptoms.

**Materials and Method:**

In the present cross-sectional/analytical study fifty subjects were selected. Clinical features and radiographic status of the teeth were also evaluated. After access cavity preparation, the retrieved material and dentinal chips removed from the root canal were transferred into 1.5-mL microtubes, followed by storage at -20ºC until used for DNA extraction. A DNeasy Tissue Kit was used to extract DNA using the DNeasy protocol for animal tissues. Master Plus SYBR Green I (Jena Bioscience, Germany) was used in a Rotor-gene Real-time PCR System for real-time PCR. The relationship between the presence of *Candida albicans* and the clinical and radiographic features were analyzed using McNemar’s test.

**Results:**

There was a significant relationship between the radiographic findings in endodontically treated teeth and the presence of *Candida albicans*. However, there was no significant relationship between the presence of *Candida albicans* and any of the clinical symptoms.

**Conclusion:**

In spite of the limitations of this study, we concluded that *Candida albicans* was associated with root canal infections in endodontic retreatment cases, but there was no relationship between root canal infections and the clinical symptoms.

## Introduction

The presence of microorganisms in the root canals is the leading cause of unsuccessful endodontic treatment [ 1 ]. Fungi have been discovered in multiple investigations [ 2 - 5 ] on endodontic infections. According to a recent meta-analysis [ 6 ], fungal pathogens prevalence in root canal infections was 9.11 percent, with 9.0% in treatment and 9.3% in retreatment cases. In research using culture techniques, this prevalence in primary and retreatment endodontic cases were 6.3% and 7.5%, while in studies using molecular techniques, it increased to 12.5% and 16.0% respectively [ 6 ]. *Candida albicans* (*C.albicans*), *Candida tropicalis*, and *Candida kefys* are the most frequently isolated fungi from infected root canals, respectively [ 7 ].

*Candida* species related infections are poorly understood in endodontics. Nonetheless, accumulating evidence suggests that the *Candida* biome is important to the pathogenesis of endodontic problems [ 8 - 10 ]. *C.albicans* has a strong affinity for hydroxyapatite and can strongly attach to untreated or ethylenediaminetetra-acetic acid/sodium hypochlorite-treated dentin [ 11 - 12 ]. The smear layer promotes *C.albicans* dentin attachment [ 15 - 16 ], most plausible as a result of the calcium ions and uncovered collagen on the dentinal surface. *Candida* can attach to collagen types IV, and I with a preference for dentinal collagen [ 17 - 20 ]. In addition, calcium ions affect Candida's capacity to proliferate and bind to proteins in the extracellular matrix [ 15 - 16 , 21 ]. *C.albicans* owns the exceptional capacity to thoroughly enter tubules owing to its thigmotropic qualities. According to these characteristics, yeast is classified as a dentinophilic microorganism [ 22 ].

Several studies also indicate that *Candida* plays a significant role in root canal related infections [ 22 - 24 ]. Being a microaerophilic eukaryote, it is metabolically adapted to live in the hard and arid root canal environment. Multiple studies have demonstrated that *in vitro*; *C.albicans* could utilize dentin as a source of nourishment colonizing both canal walls and dentinal tubules, if they did not have any other food supplements [ 22 - 24 ]. A smear layer generated by equipment helped *C.albicans* enter dentinal tubules *in vivo* [ 25 ]. Therefore, *C.albicans* protected existence within the tubules, away from equipment and irrigants, may cause endodontic infections to persist [ 17 , 26 ]. Consequently, *C.albicans* is frequently detected in endodontic infections that are resistant to root canal therapy [ 27 ]. There have also been reports of the isolation of pure *Candida* cultures from root canal infections [ 24 , 28 - 29 ].

Studies based on culture methods are a further factor to consider. If the correct culture medium is not utilized and the samples are not examined for a minimum of 3 days, the low number of fungi in the infected root canal system may take up to 3 days to proliferate as colony forming units [ 6 ]. The yeast frequency is likely to be underestimated if laboratory personnel do not use wrong culture media (such as Sabouraud agar or chromogenic agar) and/or only review data for up to 24 hours. However, using molecular mycological techniques like polymerase chain reaction (PCR) with specific universal primers can identify all kinds of fungal flora, even the uncultivable forms, regardless of the endodontic ecosystem's population size [ 6 ].

The conventional PCR method detects the presence or absence of a target microbe, but does not quantify its concentration. In addition, this approach requires post-amplification processing to segregate and identify particular PCR products [ 30 ]. Modifications to the standard PCR procedure can be used to circumvent the limitations. In the real-time quantitative PCR (qPCR) method, which incorporates the SYBR Green I system, a fluorescent dye is incorporated into the double-stranded DNA produced by DNA polymerase activity. The generation of a fluorescent signal during amplification enables the identification and quantification of products in real time [ 30 ]. There is limited data available about the relationship between *C. albicans* and endodontic symptoms, thus this study seeks to figure out the frequency of *C.albicans* in root canals of retreatment cases and its association with endodontic symptoms.

## Materials and Method

The current cross-sectional/analytical study included 50 patients referred for endodontic retreatment to the Department of Endodontics at the Tabriz Faculty of Dentistry due to reasons such as replacement of restorations and prosthesis or clinical signs and symptoms of root canal treatment failure. The study excluded patients who had taken corticosteroids or antibiotics during the preceding three months, as well as patients with systemic diseases. Additionally, teeth with more than 4 mm of probing depth and teeth that were impossible to isolate were excluded. For each tooth, the clinical manifestations of pain, sensitivity to periapical tests, sinus tracts, and swelling were documented. Using periapical radiographs taken with paralleling technique, the radiographic health of the teeth was also evaluated and recorded. Each case of periapical radiolucency or lack thereof was documented. The coronal repairs, intracanal posts, and carious lesions removed. After creating access cavities and isolating the teeth with a rubber dam, the teeth were disinfected with 5.25% sodium hypochlorite and rinsed with normal saline solution.

Root canal treatments and sample collection were conducted under aseptic circumstances. To clean out the canal filling materials, we used Gates-Glidden drills (Dentsply Maillefer, Ballaigues, Switzerland) and Hedstrom (MANI, Yohara, Japan) instead of chemical solvents. The extracted materials and dentinal chips were placed in 1.5-mL microtubes and stored at -20oC until DNA extraction.

The ATCC 36232 strain of *C.albicans* was obtained and cultured on Sabouraud dextrose agar to evaluate the procedure and the specificity of the PCR tests used in this study. Extracted DNA from the reference strain was combined with human genomic DNA in the proportions shown in Table 1. The study employed twelve different concentrations of the retrieved DNA.

**Table 1:** Control samples used

No	*Candida* standard strain (ATCC 36232) DNA (ng)	Human DNA (ng)
S1	100	0
S2	10	90
S3	10	0
S4	10	22.5
S5	1	99
S6	1	25
S7	0.1	99.9
S8	0.1	0
S9	0.1	25
S10	0.01	99.99
S11	0.01	0
S12	0.01	25

### Extraction of nucleic acids

A powdering method using liquid nitrogen was used on approximately 50 mg of the tissues removed from each tooth. The DNeasy Tissue Kit (Qiagen, Germany) was used to extract DNA from animal tissues using the DNeasy technique. 25 mg of each sample's powder was used to get the DNA, and the concentration was measured using UV absorption spectrophotometry at 260 nm (Biophotometer plus, Eppendorf, Germany).

### Oligonucleotide primers

CANAL-F (5- TTTCTCTCGCCCCGTGTGGGT-3) as well as CANAL-R (5- GGCAGCTCTACCTTCAACGCCA-3) were built by Primer3 Design software (http://frodo.wi.mit.edu/) and based on the Heat Shock Protein 60 gene, (accession number AF085694), which targets a 294 bp of mitochondrial DNA of *C.albicans*. 

A pair of primers (5'-TGTCCACCTTCCAGCAGA-TGT-3', 5'-CACCTTCACCGTTCCAGT-TTT-3') built ona chromosomally encoded ß actin gene was employed for the control amplification of a 249-bp mammalian sequence [ 31 ]. Bioneer Co., Ltd. (S. Korea) was the source of primers.

### PCR test

The conventional PCR assay was applied in a Perimus 96 thermocycling unit (Peqlab, Germany). The reaction mixture (25μL) consisted of MgCl2 (4 mM), 1 M of MdNTP mix (Bioline), 2 mM of each primer, 0.2 U Taq DNA polymerase (Bioline), and 2μL of extracted DNA. The thermocycling procedure consisted of 5 minutes at 95°C for initial denaturation of DNA and 30 cycles thereafter as follows: 95°C for 30 seconds (the denaturation phase), 60°C for one minute (the annealing phase), and 72°C for 30 seconds (the extension phase) and 10 minutes for final extension at 72°C. The traditional PCR products were separated on a 2% (w/v) agarose gel in TAE (Tris base, acetic acid and EDTA) buffer, followed by staining with ethidium bromide. A 50-bp DNA ladder (Fermentase, Vilnius, Lithuania) was utilized as a size marker. The gel photos were taken using a Syngene gel documentation system.

The real-time PCR technique was applied using Master Plus SYBR Green I (Jena Bioscience, Germany) in a Rotor-gene Real-time PCR System (Corbett Life Science, Australia). A total of 20 μL of reaction mix contained 2 μL of Master Plus SYBR Green I, 1 μL of each primer (35 nM) and 2.5 μL of the template (10 ng). In the non-template controls, the template was replaced by double-distilled water (Cinnagen Co, Tehran, Iran). The PCR cycling protocol was run as before. Concerning the melting curve, a thermal gradient was applied from 60°C to 95°C at 0.5°C/5 s. The efficacy of each reaction was determined using this formula: $E=(10^{\mathrm{(1/slope)}}-1)$. All the reactions were performed twice.

### Statistical examination

Using SAS, the data were statistically evaluated (Ver. 9.2). Using McNemar's test, the relationship between the presence of *C.albicans* and clinical and radiographic characteristics was analyzed.

## Results

Genomic DNA was detected as a high-intensity band in both the control samples (ATCC 36232) and the patient-extracted samples. Each sample’s concentration of DNA ranged between 20 and 50 g/mL.

### PCR assay

**Figure 1:**
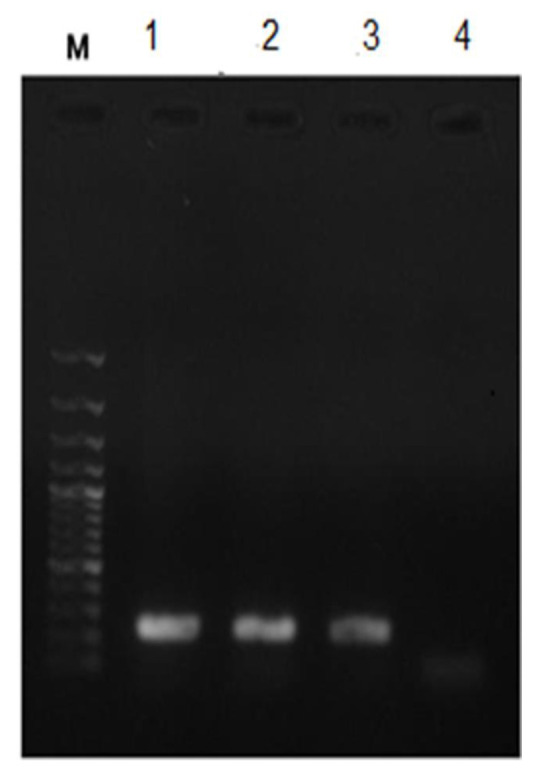
PCR amplification products with *Candida albicans* specific primer pair M: DNA ladder; 1: control sample (S1) containing 100 ng of *Candida* standard strain DNA; 2: control sample (S2) containing 10 ng, 90 ng, of *Candida* standard strain DNA and human genomic DNA respectively; 3: control sample (S5) containing 1 ng, 99 ng of *Candida* standard strain DNA and human genomic DNA; 4: control sample (S7) containing 0.1 ng, 99.9 ng of *Candida* standard strain DNA and human genomic DNA respectively

The control samples' DNA was taken out and added to the PCR mix so that it could be used as a template to test how specific the primer pair was. As expected, the control sample and four patient samples (8%) showed a specific band with 294bp (Figure 1). Containing 0.01ng *C. albicans* DNA, the PCR test came back negative (Table 1). 

**Figure 2:**
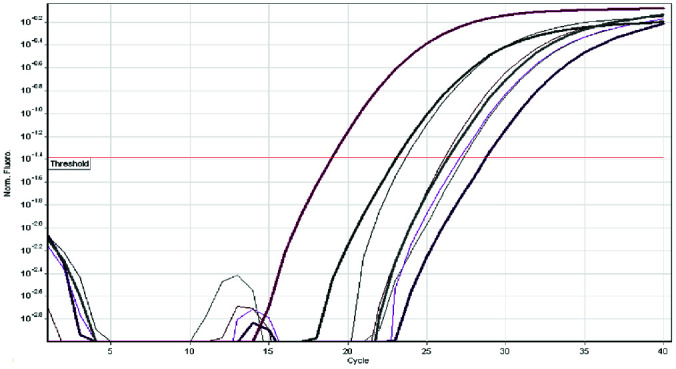
Amplification plots of SYBR green I real time PCR for a selection of control samples (thick lines) and patient samples (thin lines)

In order to quantitate, the human genomic DNA and *C.albicans* template DNA were serial diluted by 10 times, starting at 100ng/μL. SYBR Green I real-time PCR at 60°C was used to examine the primer pair (Figure 2).

**Figure 3:**
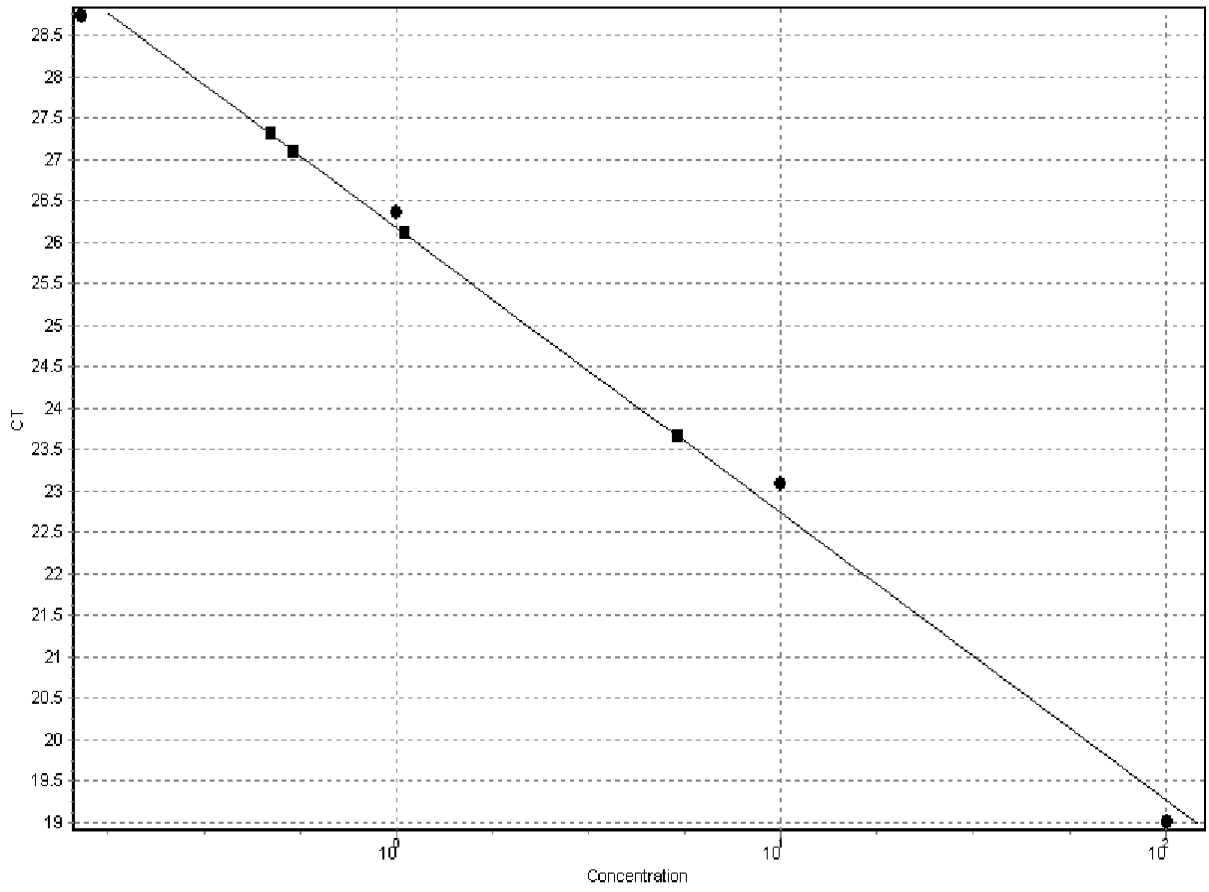
Standard curve for the SYBR green I real time PCR amplification of *Candida albicans* DNA. A plot of Ct value against the logarithm of 100, 10, 1 and 0.1 ng of extracted DNA from control samples (circles) and patient samples (squares) are indicated

**Figure 4:**
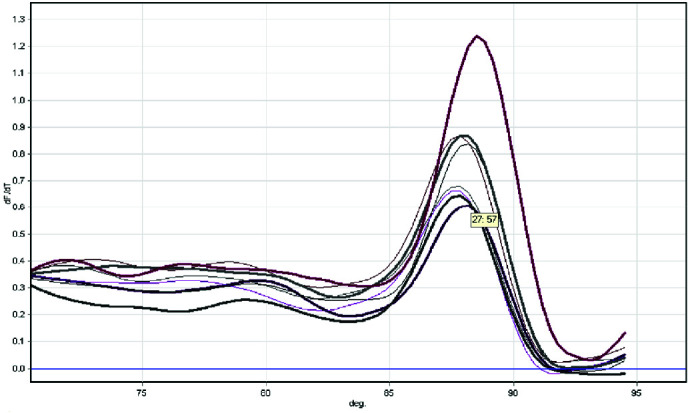
Dissociation curve analysis of SYBR green I real time PCR products of a selection of control samples (thick lines) and patient samples (thin lines)

Moreover, the formula for the threshold cycle against DNA concentration at various dilutions was y=-3.36x +28.31. The estimated regression coefficient was 0.99 (Figure 3). The response showed that there was 0.1ng of *C.albicans* DNA. The analysis of the melting curve showed a certain amplicon with a Tm value of 87.5°C (Figure 4).

After the melting curve examination, there was no peak in the control samples. Dissociation curves showed a single peak that represented the PCR product's melting point of 87.5°C. The results presented above showed no primer dimers and selective amplification. 

In 50 patient samples, four positive reactions were seen when the DNA from the study samples was added to the reaction. The positive samples had *C.albicans* DNA in concentrations as high as 4.5ng and as low as 0.42ng, respectively. The melting curve analysis of the positive samples showed the same particular amplicon. After adding various concentrations of human DNA to the extracted DNA of the *C.albicans* control specimen, the real-time PCR showed that the amount of *C.albicans* DNA was similar to what was planned. The PCR assay's effectiveness was assessed to be greater than 90%. After 36 thermal cycles, PCR with 100ng of control DNA of all the human control samples showed weak fluorescence. For the *C.albicans* control, each reaction showed 100ng of genomic DNA, with a mean Ct of 17.5.

### Relationship to the clinical manifestations

*C.albicans* was found in four (eight percent) of the fifty cases, which were the only cases with apical radiolucency. Apical radiolucency and the presence of *C.albicans* were found to have a highly significant correlation (*p*<0.0001), according to the statistical analysis. On the other hand, there was not a significant correlation found between the presence of *C.albicans* and any of the clinical symptoms, since only one of the twenty-two patients with symptoms was *C.albicans* positive (*p*> 0.05).

## Discussion

In this study, SYBR Green I Real-time PCR was used with the aim of determining the prevalence of *C.albicans* in root canals of retreatment cases and its association with endodontic symptoms. Another factor is scientists' microbiological cultivation methods, which may yield different results. If the correct culture medium is not utilized and the samples are not examined for at leastthree days, the low number of fungi in root canal may take up to 3 days to proliferate as colony-forming units. If laboratory personnel do not use the right culture media and only review culture data for 24 hours, yeast frequency may be underestimated. However, using molecular mycological techniques like PCR with specific universal primers can identify all kinds of fungal flora, even the uncultivable forms, regardless of the endodontic ecosystem's population size [ 6 ].

The conventional PCR method detects the presence or absence of a target microbe, but does not quantify its concentration. In addition, this approach requires post-amplification processing to segregate and identify particular PCR products. Modifications to the standard PCR procedure can be used to circumvent the limitations. In the real-time qPCR method, which incorporates the SYBR Green I system, a fluorescent dye is incorporated into the double-stranded DNA produced by DNA polymerase activity. The generation of a fluorescent signal during amplification enables the identification and quantification of products in real time [ 30 , 32 ].

The real-time PCR sensitivity to detect *C.albicans* was evaluated with the use of samples containing different amounts of this agent and human genomic DNA. A paper has reported sensitive TaqMan real-time PCR assays that are capable of detecting as low as 0.5 pg of *C. albicans* DNA in clinical blood samples [ 33 ]. The detection limit of SYBR Green I real-time PCR in this study was 0.1 ng, measuring *C.albicans* DNA in dental tissue mixtures.

The method used in this research was specific enough to make a distinction between *C.albicans* and human genomic DNA. The presence of different ingredients in dental tissues collected from endodontic retreatment cases, such as collagen and gutta-percha, might inhibit DNA polymerase, resulting in false negative results. We tried to minimize polymerase activity inhibition by these ingredients with the use of a silica-based method used to extract and purify DNA. 

*Candida* species endodontic infections are poorly understood. Yet, growing research suggests that the *Candida* biome is relevant to endodontic issues [ 8 - 10 ]. *C.albicans* has a strong affinity for hydroxyapatite and can strongly attach to untreated or ethylenediaminetetraacetic acid/sodium hypochlorite-treated dentine surfaces [ 11 - 12 ]. The smear layer's exposed calcium ions and collagen let *C.albicans* stick to human dentin [ 15 - 16 ]. Root canal irrigants and medicaments that have poor anti-fungal properties might promote yeast proliferation in root canals [ 27 ]. Endodontics uses calcium hydroxide predominantly intracanally. *C.albicans* thrives over a wide pH range (3.0- 8.0) and as a result, it is resistant to calcium hydroxide [ 27 ]. *C.albicans* also uses Ca2+ from calcium hydroxide for growth [ 27 ]. Thus, root canal-resistant endodontic infections commonly contain *C.albicans* [ 27 ]. 

We detected *C.albicans* in 8% of cases, and there was a significant relationship between the presence of radiolucency and *C.albicans*, consistent with other studies. Waltimo *et al*. [ 5 ] found fungi in 7% of the samples in resistant chronic apical periodontitis cases, using the culture techniques, 80% of which were *C.albicans*. Pinheiro *et al*. [ 34 ] applied advanced microbiological techniques to anaerobic species and a prevalence rate of 3.3% for *C.albicans* in root canals of endodontically treated teeth with periapical lesions. No significant relationship was found between the radiographic status of the endodontically treated and the presence of any specific bacterial species (*p*> 0.05) [ 34 ]. Ashraf *et al*. [ 35 ] studied cases of root canal retreatment, with and without periapical lesions, using a culture technique in order to evaluate the amount of *C.albicans*; 36.7% of the cases with periapical lesions had *C.albicans*, while only 13.3% of the cases without periapical lesions had *C.albicans*. There was a significant difference between the cases with and without periapical lesions [ 35 ]. These findings were consistent with those of our study; we also found a significant association between the *C.albicans* and periapical radiolucency. The coexistence of *C.albicans* and *Enterococcus faecalis* has been hypothesized to exacerbate periapical lesions by increasing the virulence of the biofilm [ 38 ]. It has been suggested that the *C.albicans* are good biofilm formers [ 36 ], and the coexistence of it with *Enterococcus faecalis* specifically accelerated osteoclastic bone resorption and inhibited osteoblastic bone growth. TNF-α and IL-6 are two examples of the inflammatory cytokines that the synergistic effects increase [ 37 ].

Dumani *et al*. [ 38 ] using PCR technique, demonstrated that *C.albicans* was present in 11% of retreated root canals with apical periodontitis in a Turkish patient group. Egan *et al*. [ 39 ] reported a prevalence rate of 16 % for yeasts in the retreatment cases with apical periodontitis, using a culture technique. Employing molecular biology techniques, researchers directly analyzed the endodontic microflora of patients from various parts of the world and discovered substantial variations in the presence of several key species [ 40 - 41 ]. Therefore, the differences in the prevalence of *C.albicans* in these studies might be attributed to geographical differences [ 42 ].

It is feasible for *C.albicans* to survive endodontic treatment and grow because of its ability to penetrate dentin, develop biofilm, and withstand routinely used intracanal antimicrobials. The likelihood of a fungal infection should therefore be considered by clinicians when retreating endodontic failures. These circumstances might necessitate further treatments, like the use of intracanal medicaments like chlorhexidine gel or alexidine digluconate [ 27 ]. In addition to having antifungal properties, recently created bioceramic sealers also have the capacity to reduce them [ 43 - 44 ]. For application in root canal obturation, novel calcium-silicate (Ca-Si) compounds that are set in wet conditions have drawn interest. They produce alkaline conditions through their hydration processes, which have antimicrobial effects, but more crucially, these materials stimulate intratubular biomineralization [ 45 - 46 ]. As root canals were filled, precipitates containing Ca-Si-based hydroxyapatite were seen inside the tubules of the dentin; these micromechanical biomineralized structures were thought to have beneficial retention [ 47 - 48 ]. It is interesting to note that additional research revealed that these biomineralized precipitates had trapped dead microorganisms inside dentinal tubules [ 49 ].

The current investigation found no evidence of a connection between endodontic symptoms and *C.albic-ans*. It might be either the nature of endodontic symptoms is multifactorial and not limited to only one organism or the current study's sample size is small. There is a limited amount of information available on this subject, thus more research is necessary.

## Conclusion

Our research indicates that the SYBR Green I real-time PCR could also be applied for molecular identification of *C.albicans* in the endodontic retreatment cases. Under the limitations of this study, we concluded that *C.albicans* was associated with root canal infections in endodontic retreatment cases. There was a significant relationship between the radiographic status of the endodontic treatment and the presence of *C.albicans*. This microorganism might be a potent pathogen in apical lesions. However, there was no significant relationship between the presence of *C.albicans* and the clinical symptoms, and more research is needed in this regard. 
